# Molecular Determinants of Cephalopod Muscles and Their Implication in Muscle Regeneration

**DOI:** 10.3389/fcell.2017.00053

**Published:** 2017-05-15

**Authors:** Letizia Zullo, Sara M. Fossati, Pamela Imperadore, Marie-Therese Nödl

**Affiliations:** ^1^Centre for Synaptic Neuroscience and Technology, Fondazione Istituto Italiano di TecnologiaGenoa, Italy; ^2^Association for Cephalopod Research (CephRes)Naples, Italy

**Keywords:** cephalopod, muscle, regeneration, development, *Octopus vulgaris*

## Abstract

The ability to regenerate whole-body structures has been studied for many decades and is of particular interest for stem cell research due to its therapeutic potential. Several vertebrate and invertebrate species have been used as model systems to study pathways involved in regeneration in the past. Among invertebrates, cephalopods are considered as highly evolved organisms, which exhibit elaborate behavioral characteristics when compared to other mollusks including active predation, extraordinary manipulation, and learning abilities. These are enabled by a complex nervous system and a number of adaptations of their body plan, which were acquired over evolutionary time. Some of these novel features show similarities to structures present in vertebrates and seem to have evolved through a convergent evolutionary process. *Octopus vulgaris* (the common octopus) is a representative of modern cephalopods and is characterized by a sophisticated motor and sensory system as well as highly developed cognitive capabilities. Due to its phylogenetic position and its high regenerative power the octopus has become of increasing interest for studies on regenerative processes. In this paper we provide an overview over the current knowledge of cephalopod muscle types and structures and present a possible link between these characteristics and their high regenerative potential. This may help identify conserved molecular pathways underlying regeneration in invertebrate and vertebrate animal species as well as discover new leads for targeted tissue treatments in humans.

## Introduction

The final functional objective of regeneration is the re-establishment of tissue after injury. This is similar—if not identical—in all species capable of regeneration. However, the mechanism through which this final goal is achieved is not entirely understood and may greatly vary among species. Recently, as underlined by Sánchez Alvarado and Tsonis (2006), a significant progress in the field of regenerative biology has been boosted due to the use of a wider range of animal models that allowed answering fundamental and common questions about the molecular basis of regeneration. As for invertebrates, regeneration is not observed in all phyla but many members of the Lophotrocozoan superphylum (e.g., polychaetes, oligochaetes, and cephalopods) display robust regenerative abilities.

Cephalopod mollusks offer a particularly viable alternative to canonical limb regeneration models due to their similarities in early arm development to vertebrate models, their complex arm structure and function, their fast and efficient regenerative capabilities and the relatively simple animal maintenance and handling (Matzner et al., 2000; Yekutieli et al., 2005a,b; Kier and Stella, 2007; Kier and Schachat, 2008; Fossati et al., 2011; Zullo et al., 2011; Tressler et al., 2014; Nödl et al., 2015, 2016).

Since a striking correlation between the muscle's ultrastructure and its physiology exists, we will attempt to describe the structure and function of the octopus musculature in order to establish the framework of where morphogenetic and more specifically regenerative processes occur in this animal species. In particular, we will highlight the main similarities and differences between a typical cephalopod striated muscle (the main muscle body constituent) and a vertebrate skeletal muscle. We will further present currently available information on the molecular pathways underlying cephalopod muscle morphogenesis during embryogenesis and regeneration.

## Cephalopods neuro-muscular system

### Comparison between cephalopod and vertebrate muscle morphology

Similar to vertebrates, cephalopod muscle cells take part in the formation of a variety of organs that dramatically differ both in structure and function. Muscle cells are present in chromatophore organs (a fundamental element of intra- and inter-specific communication), eyes, hearts, viscera, mantle, appendages (arms and tentacles), and in several other small structures.

**Figure 1:**
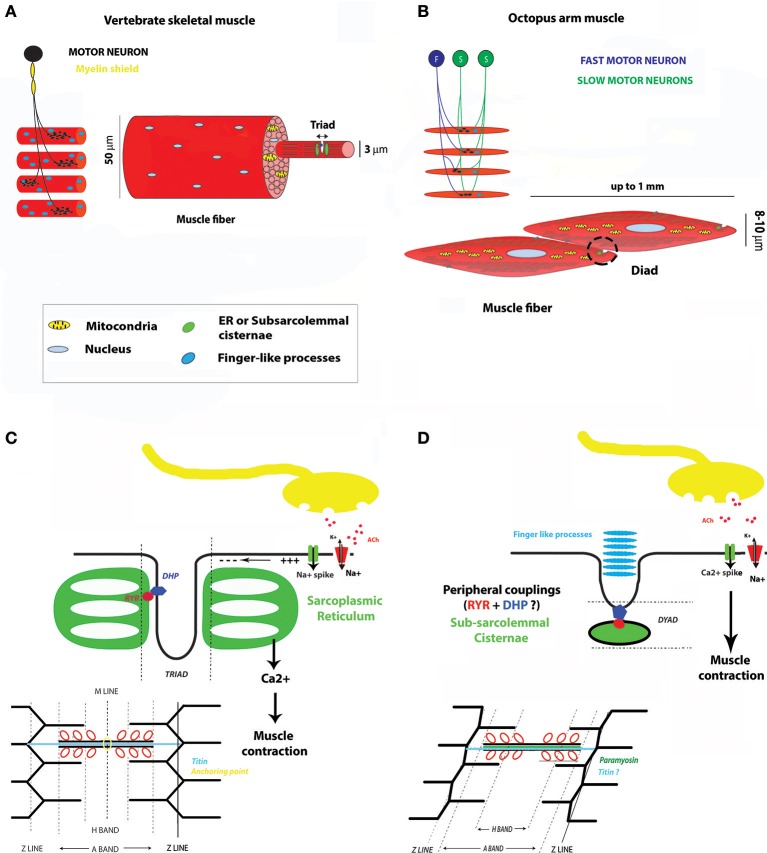
**Main similarities and differences between a vertebrate skeletal muscle and a typical cephalopod arm striated muscle. (A)** Vertebrate skeletal motor unit and myofibril. **(B)** Motor unit and muscle fiber in the octopus arm. **(C)** Vertebrate skeletal muscle at NMJ and main steps of E-C coupling. **(D)** Octopus muscle at NMJ and main steps of E-C coupling. For a better comprehension of the illustration the sarcomere was not represented at a striation angle typical to the muscle at rest (between 6° and 12°). These drawings mean to be representative of the general arrangement of muscle compartments but their single elements are not scaled on real dimensions. DHP, dihydropyridine channel; RYR, Ryanodine receptor; ACh, Acetylcholine.

As a detailed description of arm and tentacle muscle cell ultrastructure and tissue organization is reported in the companion review by Kier (2016) we will focus only on key morphological aspects essential to the comparison with vertebrate musculature (see Figure 1). Cephalopod striated muscle cells differ dramatically from musculature of known vertebrate model species. Single muscle fibers are mononucleated, at which each muscle fiber is made up by one individual cell as opposed to several fused myofibers of vertebrate skeletal muscles (reviewed in Abmayr and Pavlath, 2012). Cephalopod muscle fibers are small, generally not exceeding 8–20 μm in diameter and up to 1 mm in length, with a central mitochondrial core and a cortical zone occupied by mostly obliquely arranged myofilaments.

Within the sarcomere structure, all but the M line component are present. Filaments can show an oblique or cross striated arrangement and generally form a uniform continuous striation with adjacent muscle cells (reviewed in Kier, 2016). The nature of the filaments and associated proteins is supposedly similar to vertebrates but no direct evidence of their role in the contraction machinery has been provided so far. Interestingly, muscle genes coding for proteins involved in the contractile mechanism are expressed in both invertebrate and vertebrate developing muscles (Taylor, 1998; Carlini et al., 2000; Hooper and Thuma, 2005; Steinmetz et al., 2012). In the octopus, genes coding for Myosin heavy chain, Actin, and Tropomyosin have been proven to be expressed during differentiation of myocyte into mature myofibers (Nödl et al., 2015). Cephalopod muscle actin and myosin heavy chain, show strong sequence identity to other invertebrates and vertebrate gene ortologs (Ochiai et al., 2013; Nödl et al., 2015) while regulatory proteins, such as tropomyosin, are very cephalopod specific (Motoyama et al., 2006). As we know, differences in the amino acid sequence and structural conformation of tropomyosin have profound influence on actin affinity and are known to regulate functions of the acto-myosin activity (Hitchcock-DeGregori, 2008). In summary, this suggests that although sharing the acto-myosin composition and putatively the sliding mechanisms with vertebrate skeletal muscles, the control kinetics of the cross-bridge cycle might be different in cephalopod muscle cells.

The sarcoplasmic reticulum (SR), although not arranged to form the typical transverse tubule system (the triad) with the extracellular membrane, is present at several locations and, similar to vertebrate striated muscle, might serve as calcium storage (Kier, 1985). At the fiber border the SR forms the subsarcolemma cisternae (or terminal cisternae) that are associated with the extracellular membrane through peripheral couplings; this SR- extracellular membrane complex is also referred to as “dyads” due to its similarities to the vertebrate triad. The terminal cisternae are located at the level of each fiber's Z lines and are opposing each other in adjacent fibers. Toward the extracellular space, physical connections between muscles and the extracellular scaffold have been reported by Feinstein et al. (2011). In fact, studies using electron microscopy show the presence of finger-like processes seemingly connecting muscle cells to a collagen matrix. Interestingly, these processes are also placed at the Z lines. The localization of these two important subcellular components at the Z lines and in corresponding locations in adjacent muscle cells may suggest that the Z-discs are more than passively involved in contraction. Instead, they may also participate in mechanisms of cell signaling and stretch sensing thus acting similarly to what proposed to be a “control watch tower” for vertebrates sarcomeres (Luther, 2009). Indeed, the presence of these elements at the border of adjacent cells might account for muscle fiber coordination.

One other morphological difference between vertebrate skeletal and cephalopod striated muscles is the presence of paramyosin as an additional scaffolding protein of the thick filaments. Paramyosin molecules are packed in a crystalline array at the core of the thick filaments and bind to myosins in order to control the actin-myosin cross-bridge attachment and breaking. This process is dependent on paramyosin phosphorylation (Chantler, 1983). Kier and collaborators (Kier and Schachat, 1992) have shown that the abundance of this protein varies among different muscle fiber types, which has been correlated to the capacity of coping with increased muscle tension. The latter contributes to the specific role of each muscle type during arm movement in hydrostatic limbs such as the cephalopods arm and tentacles.

### Innervation and control of cephalopod muscles

Historically muscle cell physiology have mostly been studied in the cephalopod mantle mass and in muscle fibers of the chromatophore organs. Muscle cells in cephalopod arm musculature are isopotent meaning that each synaptic input can control the membrane potential of the entire cell via a localized synaptic junction. Muscle cells can be innervated by two kinds of excitatory nerves, the glutamatergic or cholinergic nerves, which generally produce different fiber contraction responses. Their activity was suggested to be modulated by serotonin (Bone et al., 1982; Muneoka and Twarog, 1983; Florey et al., 1985; Fox and Lloyd, 1999; Matzner et al., 2000; Rokni and Hochner, 2002). Neuromuscular synapses in cephalopod arms are exclusively excitatory and based on cholinergic innervation. Muscle cells are not multiterminally innervated, instead each one seems to be innervated at a single synaptic terminal (Bone et al., 1995; Matzner et al., 2000; Feinstein et al., 2011; see Figures 1A,B).

The electrical transduction properties of the octopus arm muscles have been extensively studied by the group of Benjamin Hochner (Matzner et al., 2000; Rokni and Hochner, 2002; Gutfreund et al., 2006). They showed that these muscles exhibit very different properties from vertebrate skeletal muscles in particular in relation to the activation/maintenance of the contraction.

Specifically, it has been shown that a single neuronal input is able to generate the special Ca^2+^ action potentials of muscle cells (see below) due to its exceptionally high quantal amplitude (5–25 mV) and fast rise time (2–4 ms). Moreover, two inputs have a low quantal amplitude (1–7 mV) and a slower time course (4–15 ms) and may thus require temporal summation to induce activation of the muscle.

Each muscle cell will then receive three types of inputs, the first two based upon small (“slow” and tonic) amplitude inputs, the third one formed by large (“fast” and phasic) non-facilitating amplitude inputs. This, together with the low density of innervation of each muscle cell, implies that the transformation of the presynaptic activity into muscle action is merely based upon a simple postsynaptic transformation mechanism (Matzner et al., 2000). The generation, frequency and duration of the spike train will then follow the size of the synaptic input with high fidelity which in turn represents a critical factor for muscle activation. At the postsynaptic side muscle cells exhibit a variety of fast regenerative responses ranging from neuron-like spikes (overshooting action potential) to fast voltage oscillations (oscillatory responses to plateau potentials; Matzner et al., 2000; Rokni and Hochner, 2002). The ionic identity underlying these action potentials is based only on Ca^2+^ spikes generated by the activation of high voltage L-type Ca^2+^ channels and followed by a rapidly activated transient of A-type K current fully inactivated in 200 ms (Rokni and Hochner, 2002). Ca^2+^ spikes cause the influx of large amounts of Ca^2+^ into the muscle cell which triggers the excitation-contraction mechanism. The role of Ca^2+^ release from the SR in octopus has yet to be elucidated but it seems clear that the first and main mechanisms of muscle fiber contraction relies upon extracellular calcium, as it is abolished when Ca^2+^ is omitted from the extracellular solution (Bone et al., 1995).

Interestingly, the somatic musculature in *Caenorhabditis elegans* (*C. elegans*) shows similar properties to that of the octopus. *C. elegans* muscle cells are uninucleated, obliquely striated, with no vertebrate equivalent T-tubule system, and rely on Ca^2+^ entry through voltage-activated L-type Ca^2+^ channels across the muscle plasma membrane to initiate muscle contraction (Waterston, 1988; Lee et al., 1997; Jospin et al., 2002). Moreover, it has been suggested that the SR is non-essential for the excitation-contraction but might be relevant to other features, such as to enhance and orchestrate the animal body motility (Maryon et al., 1998; Jospin et al., 2002). In these somatic cells the coordination of each muscle element is achieved by physiologically active gap junctions that retains small conductance properties (Phelan and Starich, 2001). Although this point has not yet been fully elucidated, in an elegant study, Liu and collaborators were able to show that these junctions are indeed responsible for muscle synchrony during *C. elegans* movements (Liu et al., 2006, 2011).

Muscle coordination in octopus might also be achieved through the presence of the terminal cisternae possibly functioning as an additional way of assuring a direct and fast contact between the depolarized extracellular membrane and the intracellular Ca^2+^ stores during muscle stimulation (see Figures 1B,D). Further investigation into this subject is required but it is worth noting that the terminal cisternae are located in adjacent cells in corresponding positions, suggesting the possibility that a certain degree of muscle ensemble coordination might rely upon them (see Figure 1B). In addition, the presence of gap junction-like structures has not been unequivocally assessed.

Several junctions have been observed in a variety of muscle cells such as those composing the stomach of Sepia and the chromatophore musculature (for a review see Bone et al., 1995). In particular, the presence of gap junctions functionally relevant for the contraction of the muscle ensemble has been demonstrated for chromatophores. Lucifer yellow staining has shown the existence of extensive dye-coupling between mantle muscle fibers in particular in squid embryos and hatchlings (for a review see Bone et al., 1995). In the octopus arm, both ultrastructural and physiological studies have failed to find indications for significant electrical coupling (Matzner et al., 2000; Feinstein et al., 2011). Nonetheless, electrophysiological experiments could not exclude the existence of low coupling coefficient between ensembles of muscle cells involved in the coordination of their activity (Matzner et al., 2000). To summarize, the main physiological differences between a vertebrate skeletal and a cephalopod striated muscle so far discovered are sketched in Figure 1.

Another intriguing analogy can be found with vertebrate cardiac muscle cells that represent an important target of regeneration medicine (Taylor et al., 2014; Kochegarov and Lemanski, 2016; Karra and Poss, 2017; Sommese et al., 2017). In these cells, Ca^2+^ currents are also at the base of spike generation, while in skeletal muscle cells spikes rely upon occurrence and propagation of Na^+^ spikes (see Figures 1C,D). However, differently from vertebrate cardiac cells, cephalopod muscle cells are able to generate a uniform change in the membrane potential of the entire cell, due to their aforementioned isopotentiality. As we will see in the next paragraph vertebrate cardiac and cephalopod striated muscle cells also share some of the genes involved in muscle formation. For instance, *NK4*, a gene essential for cardiac muscle formation in a number of metazoans, was found to be expressed in cephalopod locomotory muscle territories (e.g., arm, funnel, mantle; Navet et al., 2008; Bonnaud-Ponticelli and Bassaglia, 2014).

In conclusion, the cephalopod highly evolved nervous and neuro-muscular system can be seen as the end-point of a morpho-functional evolution toward special body dynamic requirements; this has also determined their inclusion in the EU ethical regulation (Gutfreund et al., 1996; Sumbre et al., 2001, 2005, 2006; Zullo et al., 2009, 2011; Zullo and Hochner, 2011; Fiorito et al., 2014; Berry et al., 2015; Levy et al., 2015, 2016).

In fact, the rather simple and direct transformation of neural command into muscle action and the very small motor unit volume size described above may suggest a high level of localization in the neural control of muscles and therefore a great arm movement precision which is likely to be controlled at the local peripheral level of the neuromuscular system. Interestingly this organization is optimal for feedforward motor commands that have been described to be involved in typical arm goal directed movement (Gutfreund et al., 1998; Sumbre et al., 2001, 2006).

When seen from a regenerative perspective, this highly punctual and specialized network of connections presents both advantages as well as drawbacks. In fact, in order to reconstruct a functional limb, the arm must follow a finely orchestrated mechanism of regrowth as regenerated arms are not only morphologically indistinguishable from the uninjured ones but also fully functional. The arm structure that we described here is complex in term of muscle, nerve and connective tissue architecture and has to be restored during regeneration. However, the uniform identity and innervation type of muscle cells in all muscle groups, that eventually manifests the same biophysical properties, might make cephalopod arms ideal structures for regeneration, with morphology as their major constraint.

## Muscle morphogenesis in cephalopods

### A short overview over the history of cephalopod regeneration research

The impressive abilities of cephalopods to regenerate missing or injured structures have been investigated for over 150 years following the first description of regeneration of lost appendages (i.e., the arm lost in copulation in octopods) by Steenstrup (1856) (see also Bello, 1995). Many observations and descriptions are based on specimen showing autotomy of arms and tentacles and regeneration of appendages (e.g., Verrill, 1881; Brock, 1886; Lange, 1920; May, 1933; Adam, 1937; Callan, 1939; Aldrich and Aldrich, 1968; Féral, 1978, 1979, 1988; Murata et al., 1981; Duval et al., 1984; Norman, 1992; Voight, 1992), the cornea (Dingerkus and Santoro, 1981), peripheral nerves (Sereni and Young, 1932; Sanders and Young, 1974), the shell (Meenakshi et al., 1974; Kroger and Keupp, 2004; Kroger, 2011) and even brain centers (for review see Young, 1971). These observations increased the attention on the capabilities of this taxon to regenerate several tissues and structures. Muscular degeneration and regeneration phenomena have been previously morphologically described, while the cellular mechanisms involved in the response of musculature to lesion have only started to be investigated recently. First insights into this topic were provided by Lange (1920) who illustrated the main stages of arm regeneration after injury in octopus. In this work sarcoplasm degeneration is considered as the early key event post trauma, which leads to nuclei fragmentation; sarcoblasts then migrate, contribute to blastemal formation, and proliferate to give rise to muscle fibers. More recent studies on muscle regeneration in cephalopods revealed several new and interesting aspects of the regenerative processes specifically in appendages. Arm regeneration within and across species seems to follow predictable and consistent morphological changes that lead to the restoration of full adult form and function (Lange, 1920; Fossati et al., 2013, 2014; Tressler et al., 2014; Shaw et al., 2016). These studies on the morphological processes of cephalopod arm regeneration have provided a useful basis for the examination of the molecular pathways underlying cephalopod arm morphogenesis.

### Molecular pathways underlying cephalopod muscle formation during development

The embryonic origin of muscle precursor cells, their determination to the myogenic lineage and differentiation into mature muscle cells during cephalopod development is still largely unknown. Only few studies on the embryonic formation of cephalopod musculature exist, all of which focus on the formation and differentiation of muscle tissue within the cephalopod arm crown and mantle. From an evolutionary perspective, both of these muscular structures are considered as novel adaptations of the molluscan body plan to the more active and predatory lifestyle typical for modern cephalopods (House, 1988).

**Figure 2:**
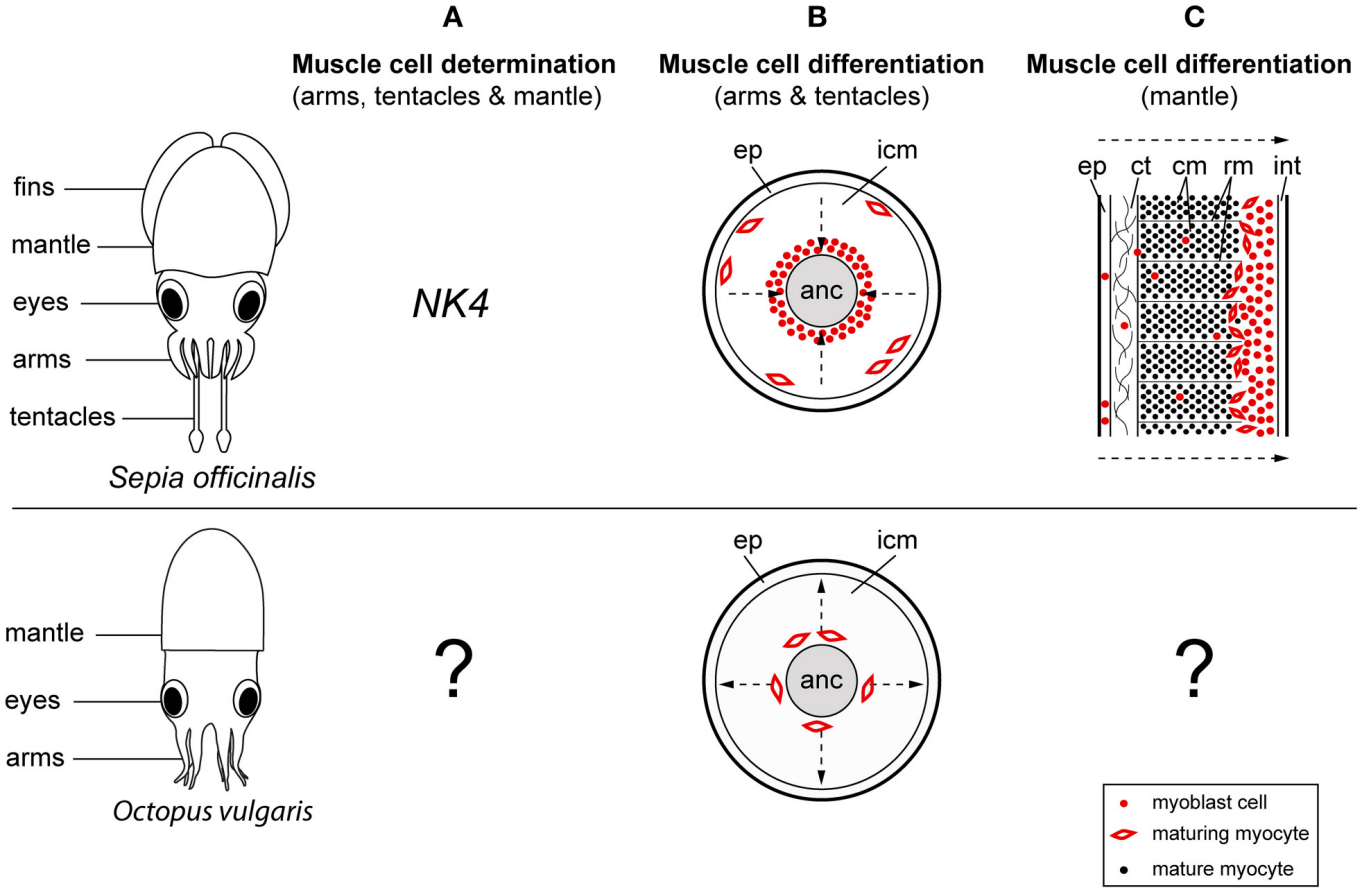
**Comparison of muscle development between the cuttlefish *Sepia officinalis* and *Octopus vulgaris***. Illustrations on the left show a cuttlefish and an octopus hatchling in a dorsal view. **(A)** *NK4* may play a role in early myogenesis of the cuttlefish while no early myoblast markers have been identified for octopus so far. **(B)** During development of the cuttlefish tentacle first mature muscle cells (myocytes) appear in the periphery of the tentacle, while in octopus first maturing myocytes are visible in the center of the arm surrounding the axial nerve cord. **(C)** The differentiation of the mantle musculature is initiated at the periphery and progresses toward the inner layers in cuttlefish. No studies have addressed this topic in octopus yet. Arrows point into the direction of muscle differentiation. anc, axial nerve cord; ct, connective tissue; cm, circular muscle; ep, epithelium; icm, inner cell mass; rm, radial muscle; int, integument.

In vertebrates, the early determination of the skeletal muscle cell lineage is regulated by the paired-homeobox transcription factors *Pax3* and *Pax7*, which subsequently initiate the expression of the myogenic regulatory factors during myogenesis (Tajbakhsh et al., 1997; Table 1). In contrast, cephalopod muscle cell determination may involve the NK-2 class homeodomain transcription factor *NK4* (Navet et al., 2008). While mostly involved in the correct morphogenesis of the heart in invertebrate and vertebrate species (Azpiazu and Frasch, 1993; Bodmer, 1993; Evans et al., 1995; Olson, 2006), NK4 is not only restricted to the prospective cardiac muscle in the cuttlefish *Sepia officinalis*. Instead transient expression was also observed within the mesodermal regions of the arm crown and the mantle primordia during early developmental stages but disappears during muscle differentiation. Navet et al. (2008) therefore suggest that NK4 may have been recruited into arms and mantle in order to participate in the early myogenesis of these novel structures (Figure 2A, Table 1).

**Table 1:** **Molecular regulation of myogenesis in vertebrates skeletal muscles and cephalopods muscles**.

	**Determination of myotome**	**Early determination of muscle cell lineage**	**Terminal specification of muscle cell lineage**	**Proliferation**	**Differentiation**
Vertebrates	Wnt, Fgf, Bmp, Shh	Pax3, Pax7	Myf5, MyoD	Fgfs	Myogenin, Mrf4, Mef2, Myf5, MyoD
Cephalopods	?	NK4	Myf5, MyoD	Hh	?

While our understanding of muscle precursor cell determination ends here, slightly more information is available on muscle differentiation in cephalopods, particularly within the arm crown. The cephalopod arm crown is a bilaterally symmetric structure, which is thought to be derived from the ventral, muscular foot of a monoplacophoran-type molluscan ancestor (Bandel and Boletzky, 1988; Boletzky, 2003; Lee et al., 2003; Shigeno et al., 2008). It consists of four pairs of prehensile arms in the octobrachian cephalopods, with an additional pair of retractile tentacles in the decabrachian, and a pair of cirri in the vampyromorph cephalopods. Prehensile arms and retractile tentacles show specific, functional adaptations, which are reflected in the different arrangement and varying striation pattern of muscle fibers between both arm types. Similarly, differences in the embryonic formation of these muscle types seem to exist.

For instance, the differentiation of muscle fibers seems to require different types of transcription factors and take place in divergent locations in tentacles and prehensile arms. In particular, during the formation of the decabrachian tentacle, myoblast cells surround the axial nerve cord in a compact sheath of cells. These migrate from the center toward the periphery and differentiate into slow, smooth-like, and fast, striated muscle cells expressing the vertebrate myoblast markers Myf5 and MyoD, respectively (Grimaldi et al., 2004a,b). Consequently, first mature muscle cells appear in the periphery of the tentacle. In contrast, during octopus arm development no expression of vertebrate-type myoblast markers was detected. Furthermore, first mature muscle cells appear in the center of the arm in the area of the future transverse muscle fibers, surrounding the axial nerve cord before the appearance of longitudinal muscle fibers in the periphery (Nödl et al., 2015; Figure 2B, Table 1). Since the homology between octopus appendages and the decabrachian prehensile arms is not entirely resolved, the observed differences in muscle differentiation may well be an octopus peculiarity. Further studies on the origin of myoblast cells and muscle development within the decabrachian arm crown will provide more insight into whether similarities between octopus and decabrachian prehensile arms exist.

Another interesting specialization of the decabrachian tentacle concerns the striation type of the decabrachian transverse muscle layer. As opposed to most cephalopod muscle fibers, which are obliquely striated, transverse muscle fibers of the tentacle show cross-striation. This specific adaptation allows the musculature to contract in higher velocity and extend the tentacle within <2 ms. Kier (1996) has shown that in the squid *Sepioteuthis lessionana* cross-striated muscle cells originate from obliquely striated muscle cells after hatching and tentacles only become functional when those specialized muscle cells are fully formed. Conversely, in *Sepia officinalis* cross-striated muscle cells appear simultaneously with obliquely striated cells during embryonic development. In fact, according to Grimaldi et al. (2004a,b) both obliquely and cross-striated cells originate from elongated myoblasts, which are positive for a vertebrate specific MyoD antibody, and give rise to the fast, glycolytic muscle fibers. However, even though cross-striated and obliquely striated cells co-exist in the hatchling's tentacles, these specialized arms similarly only become functional 2 weeks after hatching. Suggested explanations for this functional delay are an immature innervation of the musculature (Wells, 1985) or an unbalanced ratio of longitudinal to transverse muscle fibers (Grimaldi et al., 2004b).

While the general make-up of the mantle musculature is very different from that of the decabrachian tentacle, similar muscle precursor cells seem to contribute to the formation of this muscle tissue in the cuttlefish *S. officinalis* (Grimaldi et al., 2008). In particular, the early cuttlefish mantle consists of a compact mass of elongated and spherical muscle precursor cells, surrounded by a multilayered integument (Figure 2C, Table 1). This integument consists of ciliated and scattered spherical myoblast cells, which migrate through the connective tissue of the integument into the inner myoblast layers. Muscle differentiation starts from the periphery toward the inner layers of the mantle, at which elongated myocytes differentiate into radial fibers with fast, glycolytic character while spherical myocytes develop into slow, oxidative circular muscle fibers similar to the tentacle's musculature. During mantle muscle maturation radial fibers divide circular muscle fibers into parallel rows while circular musculature differentiates into the superficial slow muscle fibers (outer and inner region of the mantle) and the deeper fast circular fibers (central and radial fibers). Grimaldi et al. (2008) have further shown that during this process the proliferation and survival of myogenic precursor cells seems to depend on the signaling molecule Hedgehog (Hh) and its receptor Patched (Ptc). In vertebrates the proliferation of myoblast cells is mostly regulated by fibroblast growth factors (FGFs), but the vertebrate Hh ortholog Sonic hedgehog (Shh) is similarly required for the determination of muscle precursor cells in both trunk and limb musculature (Borycki et al., 1999; Bren-Mattison and Olwin, 2002; Table 1).

Although, similar mechanism in the early determination and formation of musculature seem to exist between vertebrates and cephalopods (Table 1), these similarities are mostly based on studies utilizing vertebrate-specific antibodies in order to identify early myoblast cells and differentiating myocytes but they have not been confirmed on the molecular level (Grimaldi et al., 2004a,b; Grimaldi et al., 2008; Bonnaud-Ponticelli and Bassaglia, 2014). However, studies focusing on species-specific gene expression (Navet et al., 2008; Nödl et al., 2015) have shown that certain features of cephalopod muscle development seem to be specific to this molluscan class. Therefore, more information will be necessary in order to understand the molecular basis of this highly complex and adapted tissue type.

### Molecular pathways underlying arm formation during regeneration

Several mollusc species are able to regenerate a variety of structures including the foot, tentacles, siphon, shell, and mantle, and even the head. Cephalopod molluscs are well-known for their capacity to regenerate their arms. However, very little data are available about this process and even less about the cellular and molecular mechanisms involved. Here we will focus on cephalopod arm regeneration process.

Regeneration in the arm begins with the process of wound healing in which the edges of the wound at the amputation site curl inward and the axial nerve cord protrudes beyond other tissues. As cephalopod blood does not clot, hemocytes and probably also muscle cells accumulates at the injured site and adhere to the plug thus closing the wound (Shaw et al., 2016).

In many animals, growth factors are fundamental to the healing processes, they activate and modulate the tissue repair process and play an important role in the formation of fibrin clots at the wound sites (Middleton et al., 2012). A proportion of growth factors are derived from blood and in particular from the platelets. Platelets are a reservoir for growth factors of various kind including platelet-derived growth factor (PDGF), epidermal growth factor (EGF), vascular endothelial growth factor (VEGF) and fibroblast growth factor (FGF).

Known growth factors in cephalopods are the EGF, FGFs, and VEGF. Their possible involvement during regeneration has yet to be assessed but evidence based on similarities with vertebrate platelet induced wound healing is in support of this. First, after amputation, cells adhering to the damaged tissue are highly interdigitated, and might be functionally equivalent to the interdigitation of platelets typical of vertebrate wounding. Second, tissue contraction around the wound is followed by active epithelial cell migration that assures the formation of the plug. Third, at the plug region a mixture of extracellular matrix (ECM), vesicles and mucus are present while fibrin elements seem to be scarce. Only in a subsequent process fibrocytes invade the region and lay down connective tissue (Wells, 1983; Shaw et al., 2016). Interestingly, in vertebrate skeletal muscles growth factors act as inducers of ECM protein synthesis and fibroblast proliferation the presence of which at the regenerating tissue is accompanied by processes of scarring and fibrosis thus inhibiting the full regeneration. Further investigations are necessary to assess the specific role and composition of the ECM at wound site in octopus. Nonetheless, the organization and fibril content of the early regenerating tip might well be modulated by growth factors controlling the level of fibrosis similarly to what happens in other animals.

Following wound healing, on a cellular level, a thin layer of undifferentiated cells appears and a mass of mesenchymal cells accumulates at the wound site forming a blastema above a highly vascularized tissue (Fossati et al., 2013). Interestingly, the activation of resident populations of somatic stem cells that proliferate to induce blastema formation has been found to be a common mechanisms of regeneration in diverse animal models (Sánchez Alvarado and Tsonis, 2006).

At the beginning of myogenesis the regenerating tip continues to grow with muscle elements strongly proliferating. At the end of arm regeneration, when the process of histogenesis occurs and the reestablishment of all the structures becomes evident, the process of cell proliferation is active mostly at the tip of the arm.

Few data are available concerning the genetic control of muscle formation during regeneration in cephalopods. Both invertebrate and vertebrate muscle development relies on Myogenic Regulatory Factors (MRF), a highly conserved family of four transcription factors: MyoD, myf-5, myogenin, MRF4 and myf-6 (Ozernyuk, 2004). However, still no data are available on their expression during muscle regeneration in octopus.

Recently, several studies in both vertebrate and invertebrate species have been pointing toward a role of acetylcholinesterase (AChE) in regeneration and development. In particular, its involvement in the regenerative process has been shown in several animal phyla such as planaria, amphibians, mollusks, insects, birds, and mammals (Singer et al., 1960; Srivatsan and Peretz, 1997; Jiang and Zhang, 2008) and there is growing evidence of the spatiotemporal regulation of its expression during early embryogenesis, neurite extension, and muscle development (Soreq and Seidman, 2001). AChE has also been found to be involved in orchestrating the formation of the octopus arm during both regeneration and development (Fossati et al., 2014).

Specifically, when arm tissue is not regenerating, AChE is mostly active in the nerve cord, where it probably exerts its cholinergic functions. In regenerating arms, AChE becomes active in several other locations such as the undifferentiated mesenchymal tissue and the newly forming musculature. In particular, during the first stages of regrowth, the activity is low and restricted to the mesenchymal tissue of the arm tip until about 20 days post injury. During the appearance of new structures such as suckers, chromatophores, muscles and nervous system, AChE activity increases strongly and active myogenesis is observed. This process lasts for 2–3 weeks after which AChE activity starts diminishing until returning back to normal levels in all tissues at the completion of regeneration.

The clear AChE localization in non-neuronal locations in regenerating arms suggests that AChE may be involved in functions other than synaptic transmission in these tissues and may play important roles in tissue morphogenesis as in other regenerating animals. In fact, AChE is a well-conserved enzyme with a variety of roles at synaptic and extrasynaptic locations. These “non-classical” roles do not appear to be directly related to catalytic properties and might not even be exerted through the catalytic site of the protein that hydrolyzes acetylcholine. These functions range from cell proliferation and differentiation to cell-to-cell interactions associated with early cell adhesion in several species (Layer and Willbold, 1995; Soreq and Seidman, 2001; Jiang and Zhang, 2008; Silman and Sussman, 2008). These results point toward the existence of molecular pathways involved in regeneration, which seem to be conserved between cephalopods and known animal models and may open up the possibility to use invertebrates, such as cephalopods, as model systems for regeneration in translational medicine.

To accomplish this aim, the next fundamental step would be to perform cell lineage analysis of regenerating arms in order to determine cell type, position and specificity within the reconstructing tissue complex. Thereby, we will be able to assess the existence of pluripotent vs. lineage-committed progenitor cells, as well as vertebrate satellite-like cells associated with adult muscles.

## Summary and perspectives

Regeneration is a complex process which involves a number of cellular mechanisms depending on the animal's phylogenetic position and the tissues developmental constraints (Tiozzo and Copley, 2015). Due to the great diversity among metazoan animal species it seems challenging to find common mechanisms involved in the regenerative process. However, commonalities may exist within the phylogenetic framework. Studying the ability of muscle tissue to regenerate is particularly interesting as most vertebrate muscle genes and proteins have invertebrate homologs, which must interact correctly in order to perform relatively similar functions (Hooper and Thuma, 2005; Hooper et al., 2008; Søvik and Barron, 2013). Therefore, invertebrate muscle genes and proteins may reveal general principles that could be applied to other animals as well as to humans.

This review aimed at providing an overview on how cephalopods can be utilized to answer questions on muscle regeneration, which are equally as important to vertebrate species. Using a bottom up approach, we showed similarities in the basic architecture of the contractile machinery in octopus striated and typical vertebrate skeletal muscle. However, differences to other animal species do exist within the individual proteins of the cephalopod contractile machinery. In fact, evolution seems to have privileged the conservation of “core” structural proteins (such as actin and myosin) determining the mechanism of muscle cell contraction in spite of proteins (such as tropomyosin) involved in the fine regulation of its kinetics.

We then reviewed the “mechanistic” events and known molecular pathways underlying limb morphogenesis in embryonic development and adult regeneration in order to find a possible point of convergence with that of other animal phyla. We show that cephalopods seem to share the morphology of early stages of regeneration with other animals based on the formation of a blastemal like region of undifferentiated cells, abundant vascular components and low fibrillar ECM matrix. The possibility that these factors affect tissue competency to regrowth is one of the many interesting open questions. Vertebrates skeletal muscles are limited in their ability to regenerate and they show scarring and fibrosis characterized by the accumulation of fibril elements upon injury. These processes might intrinsically restrict the muscles complete recovery of function (Middleton et al., 2012) as a high amount of physical barriers for intercellular communication and tissue remodeling can negatively interfere with regeneration (Jazwinska and Sallin, 2016). While this is one of the limiting factor of the vertebrates ability to regenerate, cephalopods might have evolved a fine mechanisms of regulating ECM composition and organization during regeneration possibly also through the involvement of locally active growth factors.

At a molecular level several components involved in the formation and regeneration of cephalopod arm musculature seem to be conserved between cephalopods and vertebrate species. Yet, individual transcription factors seem to have been recruited specifically for the formation of musculature in cephalopods (e.g., NK4).

It is still unclear how our current understanding on cephalopod muscle physiology, development, and regeneration fits into the phylogenetic framework. However, an evolutionary robustness seems to exist which preserves basic cell components of the muscle system (such as the acto-myosin complex) but allows for a well-refined species-specific control mechanism. Regenerative ability may therefore have evolved together with the cellular capacities and constraints acting from gene to morphology and function on any level of animal complexity. Whether and how the regenerative capabilities are linked to structural differences in muscle composition is debatable but would be an interesting question to tackle in the light of animal evolution (Katz, 2016).

Although all of the above mentioned areas of study still require more detailed examination, we believe that the current effort in cephalopod research has the potential to find divergent as well as conserved morphological patterns and molecular pathways which might be activated in a number of animal species to promote regeneration. Cephalopods, and more generally invertebrates, may therefore offer an opportunity to act as stepping stones for understanding capabilities of metazoan regeneration.

## Author contributions

All authors contributed to writing this review. Specifically, LZ wrote for the paragraph “Cephalopod neuro-muscular system,” PI wrote the paragraph “A short overview over the history of cephalopod regeneration research,” MN wrote the paragraph “Molecular pathways underlying cephalopod muscle formation during development,” SF wrote for the paragraph “Molecular pathways underlying arm formation during regeneration.” LZ and MN proof-read the entire text.

### Conflict of interest statement

The authors declare that the research was conducted in the absence of any commercial or financial relationships that could be construed as a potential conflict of interest.

## Abbreviations

AChE: Acetylcholinesterase
EGF: epidermal growth factor
ECM: extracellular matrix
FGF: fibroblast growth factor
Hh: hedgehog
MRF: myogenic regulatory factors
Ptc: patched
PDGF: platelet-derived growth factor
PSP: postsynaptic potentials
SR: sarcoplasmic reticulum
Dhh: sonic hedgehog
VEGF: vascular endothelial growth factor.
